# Wild and Domestic Pig Interactions at the Wildlife–Livestock Interface of Murchison Falls National Park, Uganda, and the Potential Association with African Swine Fever Outbreaks

**DOI:** 10.3389/fvets.2016.00031

**Published:** 2016-04-14

**Authors:** Esther A. Kukielka, Ferran Jori, Beatriz Martínez-López, Erika Chenais, Charles Masembe, David Chavernac, Karl Ståhl

**Affiliations:** ^1^Center for Animal Disease Modeling and Surveillance (CADMS), VM: Medicine & Epidemiology, University of California Davis, Davis, CA, USA; ^2^Integrated Animal Risk Management (AGIRs), CIRAD Campus International de Baillarguet, Montpellier, France; ^3^Department of Animal Science and Production, Botswana University of Agriculture and Natural Resources, Gaborone, Botswana; ^4^Department of Disease Control and Epidemiology, National Veterinary Institute (SVA), Uppsala, Sweden; ^5^Department of Biomedical Sciences and Veterinary Public Health, Swedish University of Agricultural Science (SLU), Uppsala, Sweden; ^6^Department of Biological Sciences, Makerere University, Kampala, Uganda; ^7^Control of Exotic and Emerging Animal Diseases (CMAEE), CIRAD Campus International de Baillarguet, Montpellier, France

**Keywords:** African swine fever, bushpig, warthog, interface, interactions, Uganda

## Abstract

Bushpigs (BPs) (*Potamochoerus larvatus*) and warthogs (WHs) (*Phacochoerus africanus*), which are widely distributed in Eastern Africa, are likely to cohabitate in the same environment with domestic pigs (DPs), facilitating the transmission of shared pathogens. However, potential interactions between BP, WH, and DP, and the resulting potential circulation of infectious diseases have rarely been investigated in Africa to date. In order to understand the dynamics of such interactions and the potential influence of human behavior and husbandry practices on them, individual interviews (*n* = 233) and participatory rural appraisals (*n* = 11) were carried out among Ugandan pig farmers at the edge of Murchison Falls National Park, northern Uganda. In addition, as an example of possible implications of wild and DP interactions, non-linear multivariate analysis (multiple correspondence analyses) was used to investigate the potential association between the aforementioned factors (interactions and human behavior and practices) and farmer reported African swine fever (ASF) outbreaks. No direct interactions between wild pigs (WPs) and DP were reported in our study area. However, indirect interactions were described by 83 (35.6%) of the participants and were identified to be more common at water sources during the dry season. Equally, eight (3.4%) farmers declared exposing their DP to raw hunting leftovers of WPs. The exploratory analysis performed suggested possible associations between the farmer reported ASF outbreaks and indirect interactions, free-range housing systems, dry season, and having a WH burrow less than 3 km from the household. Our study was useful to gather local knowledge and to identify knowledge gaps about potential interactions between wild and DP in this area. This information could be useful to facilitate the design of future observational studies to better understand the potential transmission of pathogens between wild and DPs.

## Introduction

During the last few decades, a better understanding of how wildlife–livestock interactions potentially contribute to infectious disease emergence has led to an increase of interest on this topic (1). The opportunities for such interactions to occur have escalated due to the expansion of human population and subsequent encroachment into wildlife habitats (2). Indeed, human population growth is expected to reach nine million people by 2050 (3), leading to an ever-increasing request of animal protein (4, 5), consequent continuation of agricultural land expansion, and more opportunities of contacts between wildlife and humans.

More than 70% of the emerging zoonotic infectious diseases originated during the last decades are thought to be of wildlife origin (6). Wildlife can act as reservoir of several diseases and therefore foster spill-over events in naive or non-infected livestock populations (1). Some examples could be tuberculosis infection in South Africa (with buffalo and cattle populations in the spotlight) (7) and foot-and-mouth disease maintenance at the Great Limpopo Transfrontier Conservation Area (buffaloes being the major reservoir of infection) (8–11). This potential transmission of diseases at the wildlife–livestock interface can have a huge economic impact due to trade restrictions, losses in animal production, and the need of implementing expensive preventive programs or more drastic control and eradication interventions (i.e., vaccination or culling) (12, 13). As a consequence, a better understanding of wildlife–livestock interactions is crucial to better comprehend the eco-epidemiology of diverse pathogens affecting both wildlife and livestock and to implement more cost-effective preventive and control strategies (14).

In Uganda, the total number of domestic pig (DP) has been increasing steadily since early 2000, reaching a census of 3.7 million in 2014 (15). Under these circumstances, swine diseases are of increased interest in the pig industry. One of the diseases that has been recognized as a major constraint to the development of the pig industry is African swine fever (ASF). ASF virus is a DNA arbovirus that affects both wild and domestic swine, causing devastating economic losses in pig production. Historically, the virus circulated between soft ticks from the *Ornithodoros moubata* complex and warthog (WH), in what is known as the sylvatic cycle. Today, a domestic cycle with DP to DP transmission is believed to be the most important route of virus dissemination (16). In Uganda, the virus circulates both in the sylvatic cycle and the domestic cycle, but the understanding of the interface between these cycles, and the nature and frequency of interaction between DP and wild pigs (WPs), is limited. On the other hand, in other areas of the continent such as Southern Africa, the link between the cycles has been well described (17, 18).

In WHs (*Phacochoerus spp*.) and bushpigs (BP) (*Potamochoerus spp*.), the virus causes an asymptomatic infection. WH and soft ticks are considered to be the natural reservoirs that maintain the infection in the environment (18, 19). To this date, WHs are only known to transmit the virus through tick vectors (20). In contrast, experimental studies suggest that BP can transmit ASF virus by direct contact to susceptible DP (21). Some studies have suggested a potential role of the BP in the epidemiology of ASF (22), but to the best of our knowledge, prevalence levels of ASF in BP populations, the level of interaction between BP and DP in natural settings, and the potential risk of ASF transmission into the DP value chain given any interaction has not been investigated to date (17, 23). Thus, it remains unknown whether BP contribute to the maintenance of the disease in the environment and to the spread of ASF virus into the DP value chain. Currently, major information gaps remain in this field, particularly related to the ecology of wild African pigs and their role in disseminating infectious diseases among DPs.

Interactions between wildlife and livestock have been assessed in different settings through different methodologies, such as telemetry (24, 25), camera traps (26), the use of potential biomarkers (27), or the collection of local knowledge through interviews. The later method has been used in different settings in Africa and developed countries (10, 28–30) and is considered a practical, fast, and adequate approach to gather preliminary information on interactions between wild and domestic animals (8, 9).

The general purpose of this study was to collect local knowledge on the interactions between wild and DPs in order to better understand the potential role of African WP in the dissemination of infectious diseases at the wildlife–livestock interface. The main objective was to investigate the nature, frequency, duration, and distribution of direct and indirect interactions between WP and DP, and the associated human behavior and husbandry practices susceptible to affect those interactions, at the northern edge of Murchison Falls National Park in northern Uganda. Additionally, we also aim to evaluate the potential association between these WP–DP interactions and ASF outbreaks reported by farmers.

## Materials and Methods

### Ethics

Permission to carry out the study was granted by the Ugandan National Council for Science and Technology under the reference number A497. The Nuremberg Code was followed. A written consent from the District veterinary officer was obtained prior to the start of any activity in the area. At the time of the interviews, participants were informed that the study was voluntary, confidential, and that they had the choice of ending their participation at any time. All subjects gave written informed consent in accordance with the Declaration of Helsinki (see Data Sheets S1 and S3 in Supplementary Material: consent form and PRA consent form).

### Study Area

**Figure 1:**
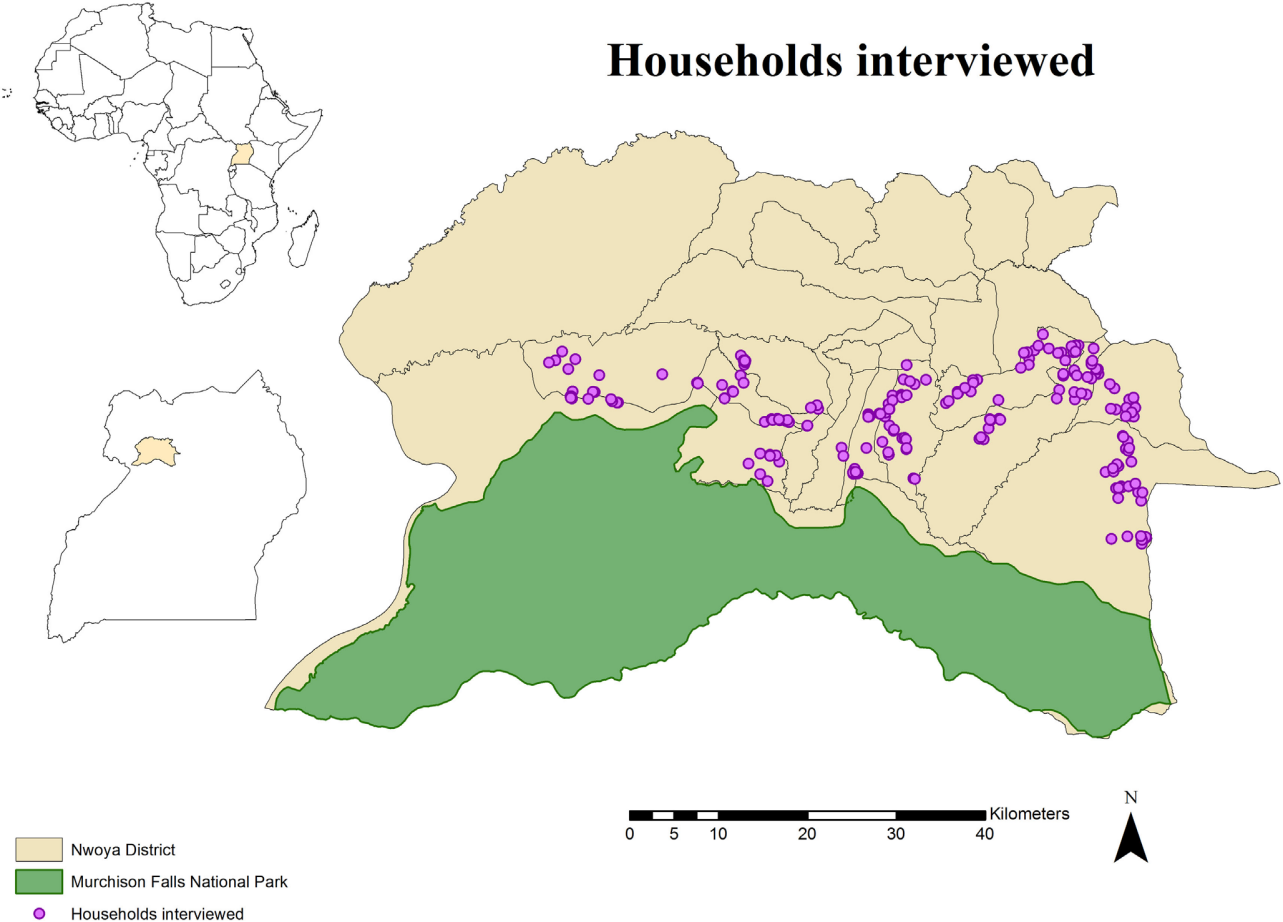
**Spatial distribution of 233 pig owning households individually interviewed in southern Nwoya district, Uganda, near Murchison Falls National Park, 2015**.

The study area comprised the northern boundary of Murchison Falls National Park and the adjacent rural communities in northern Uganda. Specifically, the study was carried out in 24 villages of the southern parishes (*n* = 10) of Nwoya district (total population: 138,500; area: 4,736 km^2^), an administrative unit in the Acholi subregion of northern Uganda (Figure 1). The subregion has a tropical climate with a rainy season from April through November and a dry season from December to March. The area was strategically selected for this study due to recurrent ASF outbreaks in a growing free-range DP population (15, 31–33), and its proximity to an unfenced national park where BP and WH are known to be abundant. These circumstances offer a suitable area for WP and DP interactions to occur.

#### Individual Interviews

##### Sample Selection

The study population for the individual interviews included households (HHs) rearing DP at any point during 2014. A census was created by consulting key informants and the chairman of the local council at the same time that a short explanation about the project was presented to them. A total of 357 HHs were identified. Because it was assumed that no husbandry differences exist within Nwoya district, a total of 233 HH were selected from the initial census using a simple random sampling approach in EpiTools^1^ (Figure 1). This number was obtained assuming a probability of 50% to observe WP and DP interactions (due to the lack of previous information) and an expected sensitivity of 80% (based on the assumption that respondents would not recall the exact number of WP seen during the previous year), a specificity of 95% (based on the assumption that most of respondents would be able to differentiate WP from other animals), a confidence level of 95%, and a desired precision of 0.05. A replacement list was also created from the remaining candidate HH (*n* = 124) in case originally selected HH were not available for interviews.

##### Questionnaire Design

The questionnaire for the individual interviews was written in English, reviewed by a local and international team of veterinarians and scientists, and uploaded in the KoBo toolbox (KTB) online platform^2^ (Data Sheet S2 in Supplementary Material: individual questionnaire). Interviews were administered in the local language, Luo, by a trained facilitator fluent in both Luo and English. Responses and HH geolocations were collected using a tablet device (Nexus 9, HTC Corp.). The trained facilitator was accompanied by the first author of this study to supervise and clarify any questions. In order to evaluate the understanding and appropriateness of the questions by all the stakeholders, a pilot trial of seven HH was carried out in the vicinity of the study area. Changes in the questionnaire were made accordingly before the start of the study. The questionnaire was divided into four sections (Table 1). The first two sections inquired about direct and indirect interactions, and the remaining sections asked about suspected ASF outbreaks, husbandry practices (i.e., housing system used), and human behavior (i.e., if anyone in the house hunted WP, management of infected carcasses, and offal). A direct interaction (DI) was defined as the simultaneous presence of WP and DP (i.e., seeing both species using the same space, at the same time) within an area of the size of a football pitch, as used in other similar studies (10, 34). An indirect interaction was defined as the asynchronous presence (i.e., seeing both species using the same space, at different time) of WP and DP in the same area (an area of the size of a football pitch). We assumed that DP could roam throughout the villages under study (35); thus, we considered any place where WP had been seen – within the village area – as a potential spot for an indirect interaction with DP to happen. An ASF outbreak was defined as the respondent’s perception of having suffered an ASF outbreak in his farm, without any laboratory confirmation. Outbreak definition based on farmer’s experience was founded on the work done by Chenais et al. (32), which showed that most farmers in the area have adequate knowledge of ASF clinical signs, and Chenais et al. (33), which suggests that farmer self-reports of ASF in our area of study are valid and accurate. The outbreak definition based on farmer’s experience was also supported by the work of Muhangi et al. (36), which upholds the idea that other DP diseases compatible with ASF clinical signs (such as classical swine fever and porcine reproductive and respiratory syndrome) are very rare or absent in Uganda. A WH burrow was defined as “active” when it was used by WH during the study period (i.e., 12 months: March 2014–February 2015), according to the respondents.

**Table 1:** **Main variables collected during individual interviews implemented in household-owning pigs at Nwoya district, Uganda, in 2015**.

**Indirect interactions**	WP presence in the village WP proximity to the household Number of WP seen in the village WP sightings seasonality WP sightings location Presence of WH burrows near the village
**Direct interactions (DI)**	DI occurrence DI location, duration, type^a^, and seasonality Distance between species during a DI Nuisance from DI Measures taken to avoid DI
**Household demographics and characteristics**	Age class of pigs present at the homestead at the moment of the questionnaire Housing system(s) used during the year Water access to pigs WP hunting habits (bag number per month, location of WP slaughtering, and offal management)
**Disease information**	ASF outbreak suspicion ASF outbreak seasonality Pig causalities and resistance Carcass management Presence of WP around carcasses

*Type of direct interaction^a^: mating/courtship, fighting, eating together, and drinking together*.

##### Questionnaire Implementation

Individual interviews were held between March and April 2015. Chairmen of the Local Councils and respondents who owned a mobile phone (*n* = 57) were contacted 2 days before the administration of the questionnaire to inform the rest of the participants about the upcoming visit to their village. In case no phone number was available, the facilitator would visit the chairmen to indicate the names of the participants to be mobilized and interviewed. Interviews were carried out at the respondent’s HH. Respondents who were unavailable at the time of the interview were contacted a second time or substituted by the first HH in the replacement list from that same village. Chairmen were invited to participate as auditors in their respective villages.

An expanded explanation of aims and limitations of the study, including a consent form (Data Sheet S1 in Supplementary Material: consent form), was given to the participants and chairmen both verbally and in written form before the start of the interviews. Participants were offered the possibility to ask questions related to the project and to ASF. In order to ensure the correct identification and to reduce misclassification of WH and BP, laminated pictures of both species were shown to the respondents when the appropriate questions were asked. At the end of the questionnaire, participants received a deworming tablet for each of their pigs as compensation for participating in the study.

#### Participatory Rural Appraisal

A series of participatory rural appraisals (PRAs) (*n* = 11) was implemented as a triangulation method to cross-verify data gathered at the individual interviews regarding WP and DP interactions. The approach was based on Chenais et al. (32) and was carried out during 4 days during June 2015. Briefly, the participants were chosen following a purposive sampling strategy in which the selection criterion was having answered in the individual interview that they had observed WP in their area. Participants were informed by key informants and chairmen in the same manner as detailed for the questionnaires. Respondents were allocated to specific dates and venues in order to maximize their commute efficiency. All villages of the study area were included. A written consent to participate in the study was collected from each of the respondents. Respondents kept a copy of the consent for reference (Data Sheet S3 in Supplementary Material: PRA consent) and received a small monetary compensation for transport expenses.

Survey tools included seasonal calendars, listing, and hand count (37). The exercise was divided in two sections: seasonality of several factors (rainfall, WP’s hunting, presence of WP in the community, and crop damage) and questions related to DIs between WP and DP (Data Sheet S4 in Supplementary Material: PRA questions). Once the PRA exercise was over, a 30-min basic training on ASF was offered as requested by the questionnaire’s respondents.

### Statistical Analysis

Descriptive analyses and data visualization using graphs and maps were conducted in R software version 3.1.0 (38), plotly (39), and ArcGIS (40) for the data collected through the individual interviews. Summary statistics, including measures of central tendency and dispersion, were computed for all collected variables. The *T*-test was used to assess differences between season and ASF occurrence. Spearman rank correlation coefficients were used as measure of association between the number of ASF outbreaks and WP sightings and housing system. BP and WH reported that the number of sightings per square kilometer was visualized using the kernel density algorithm implemented in ArcGIS (ESRI^®^) using a bandwidth of 10 km.

The relationship between collected variables and ASF occurrence was assessed using a non-linear multivariate approach referred to as multiple correspondence analysis (MCA). The MCA was conducted as an exploratory or descriptive graphical method to identify categorical variables correlated among each other (41, 42). This analysis facilitates the visualization of such correlations by displaying each variable as a point in a multidimensional Euclidean space. MCA can be considered an extension of correspondence analysis adapted for more than two variables (43, 44). The output of the MCA was created following the analysis of a multidimensional contingency table (Burt table) that was further analyzed to measure the variance of dispersion of its components in a pairwise manner (41). In order to group together individuals with similar characteristics and to identify the most indicative variables of each of the groups (or clusters), a divisive hierarchical clustering analysis (HCA) was computed. The number of clusters was decided by visual inspection of the overall appearance of the hierarchical tree and the visual representation of the individuals in the first two dimensions (45). Ward’s criterion was used to create the hierarchical tree (46). FactoMineR package (47) in R was used for both the MCA and the HCA.

## Results

From the 233 originally selected candidate HH, 214 participated in the individual interviews, and the remaining 19 participants were obtained from the replacement list. A total of 11 groups with five to seven participants per group from two to seven villages participated in the PRA (*n* = 62, number of participants). All selected candidates agreed to participate in the study.

### Interactions between Wild and Domestic Pigs

#### Individual Interviews

**Figure 2:**
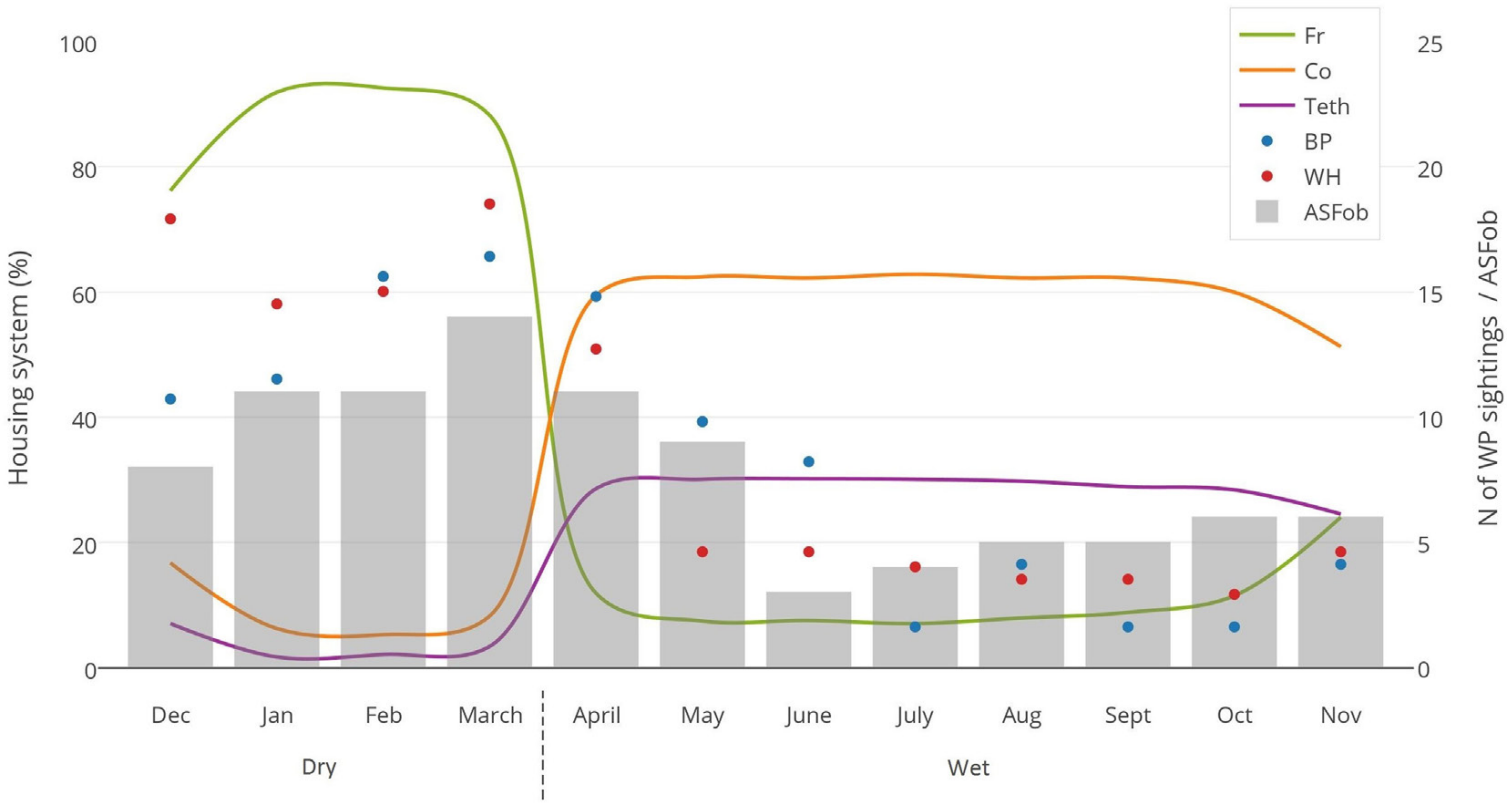
**Monthly evaluation of three variables collected during individual interviews with 233 pig owning household in Nwoya District, Uganda, in 2015**. Lines represent the type of housing system used (Fr, free range; Co, confined; Teth, tethered); dots represent the number of wild pig sightings (BP, bushpig; WH, warthog); and bar charts represent the number of ASF outbreaks (ASFob, ASF outbreaks) during the dry and wet seasons.

**Figure 3:**
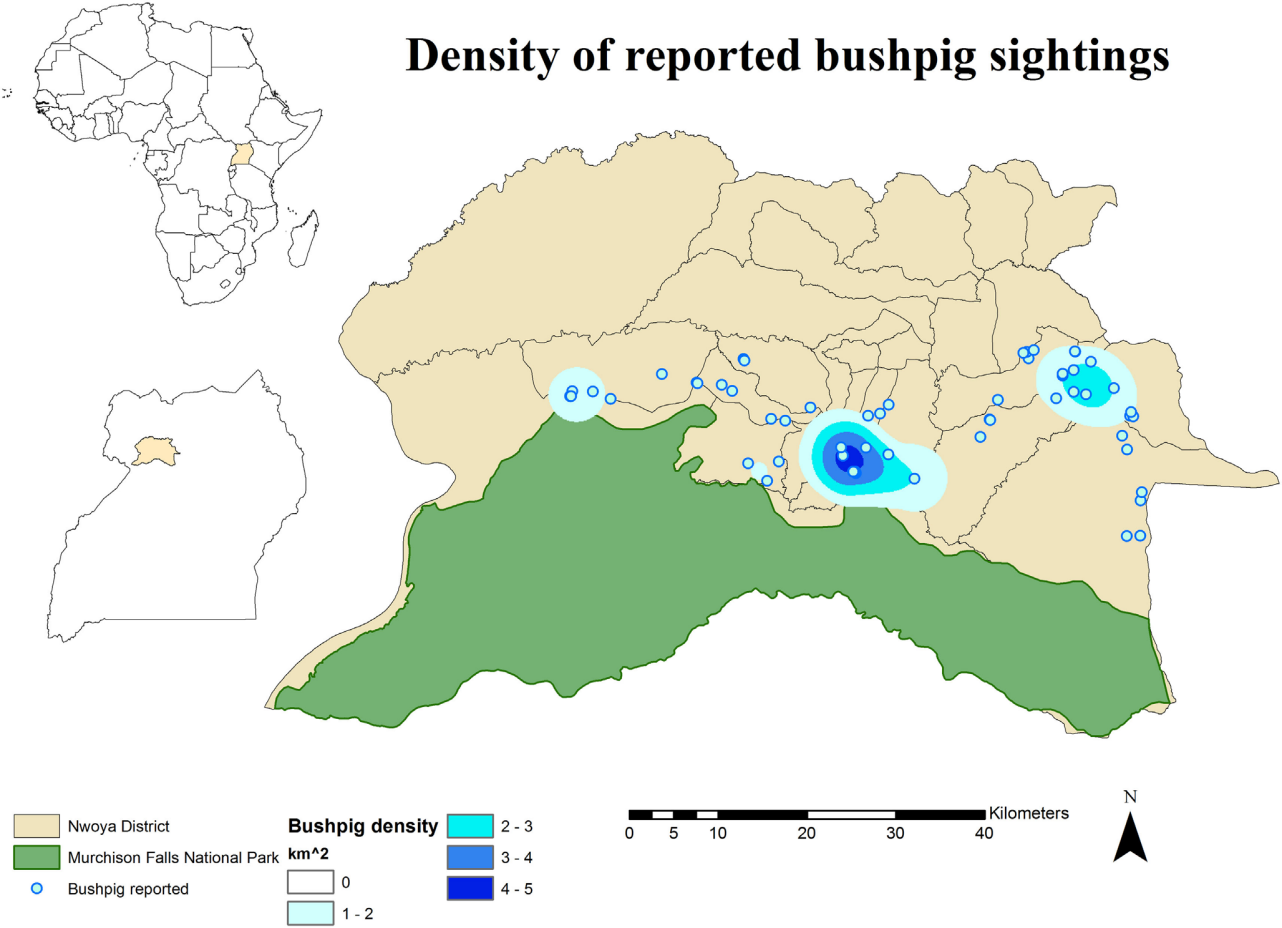
**Spatial distribution of 233 pig owning households individually interviewed in southern Nwoya district, Uganda, near Murchison Falls National Park, 2015 and kernel density estimation of bushpig sightings distribution as reported by the interviewers**.

**Figure 4:**
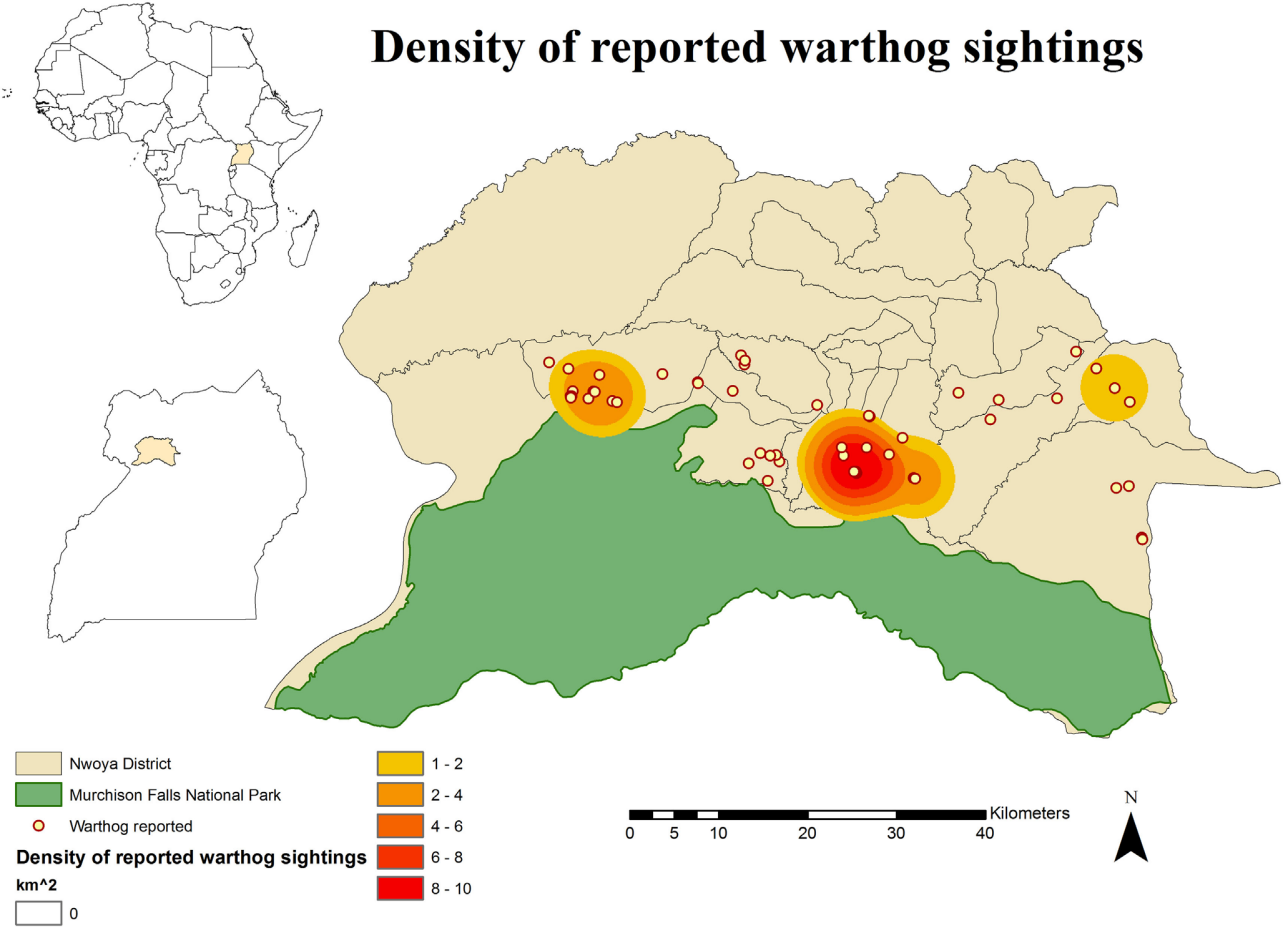
**Spatial distribution of 233 pig owning households individually interviewed in southern Nwoya district, Uganda, near Murchison Falls National Park, 2015 and kernel density estimation of warthog sightings distribution as reported by the interviewers**.

No DIs between any of the WP species and DP were reported by any of the individually interviewed participants. On the other hand, the proportion of respondents having seen indirect interactions during the previous 12 months was 35.6% (*n* = 83/233), whereas another 6.9% (*n* = 16/233) had seen the footprints, but not the actual animal. Observations of WP were reported all year round, but a higher number was reported during the dry season (Figure 2). BP and WH were often observed (indirect interactions) in swampy areas (47 and 33% of their sightings, respectively), while WH were also frequently observed in savannah-bush areas (49% of its sightings) (Table 2). Active WH burrows in the area were seen by 23.6% (*n* = 55/233) respondents, out of which 60% (*n* = 33/55) were reported to be located less than 3 km away from the respondent’s HH. The distribution of WH indirect interactions as reported by farmers was gathered in areas closer to the park, whereas those of BP tended to be more evenly distributed near HH settlements (Figures 3 and 4).

**Table 2:** **Number of indirect interactions between domestic and wild pigs per location, described during individual interviews implemented in household-owning pigs at Nwoya district, Uganda, in 2015**.

	Savannah-bush	Swamp	River	Cassava	Corn	Peanut
**Bushpig**	12 (15.8)	36 (47.4)	2 (2.6)	15 (19.7)	3 (3.9)	8 (10.5)
**Warthog**	28 (49.1)	19 (33.3)	1 (1.8)	6 (10.5)	2 (3.5)	0
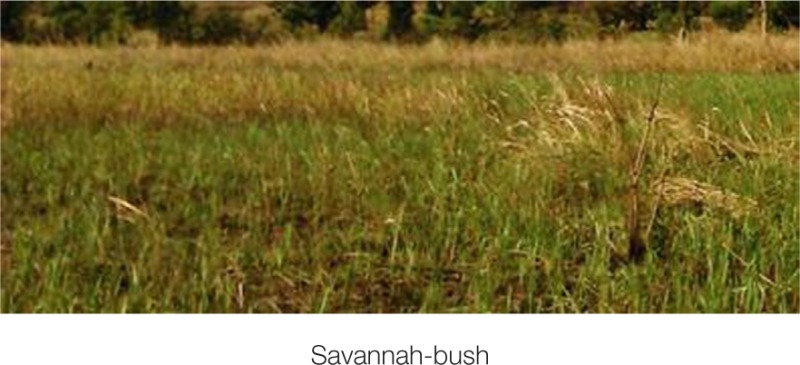				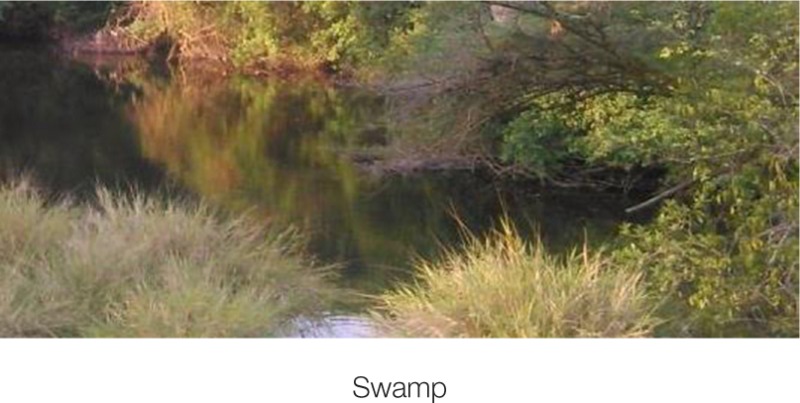		

*The percentage of sightings per species in each of the locations is depicted in parenthesis. Details of the two most mentioned areas (swamps and savannah-bush areas – i.e., plains characterized by coarse grasses and scattered tree growth) are depicted by photographs*.

Most respondents (88%) confined or tethered their DP during the wet season and left them on free range during the dry season (Figure 2). Only 38 (16.3%) respondent’s HH reported to have private sources of water close to the HH (<500 m). Most HH (*n* = 195, 83.7%) provided a mix of natural and private water sources for which DP had to walk >500 m from their respective HH to have access to it. A total of 37 respondents (16%) declared being WP hunters. In those HH, the hunting bag during the dry season (*n* = 76 WP) was more than double when compared to the one reported in the wet season (*n* = 34 WP) (*p* = 0.097). Thirty-six hunters slaughtered WP at the hunting site, while one slaughtered at home. Regarding carcass management, 30 hunters declared leaving the carcass’ offal in the field, 5 gave it to their dogs, and 2 ate all parts of the carcass. Eight hunters (3.4% of the 233 respondents) fed their DP with raw offal from WP carcasses.

#### Participatory Rural Appraisal

Participatory rural appraisal results supported those obtained through individual interviews, providing an indication of consistency in farmer’s reports. None of the groups (*n* = 11) reported having seen or heard about any DI between WP and DP. Reasons given for such were, e.g., that “they have a different scent, which makes them stay far apart” (three groups), “they fear each other” (two groups), and “WP mostly move at night, unlike DP” (one group). Hunting of both WP species was reported to be more frequent during the dry season. PRA respondents stated that the shorter length of the grass during the preparation of the crop fields facilitated the observation and hunting of WP. One group attributed the smaller hunting bag during the wet season to the fact that there was less time for hunting due to agricultural activities. BP was described as an elusive animal. PRA respondents reported that this species came closer to the communities during the wet season when the grass was higher (which offers protection) and crops were available. One group reported seeing BP in January, when they believed BP approached the community in search for water sources. Conversely, WHs were mainly reported to be spotted during the dry season, although crop damage was reported throughout the year (with a decrease from March to May). Respondents believed that WH thrived in open areas and disliked high grass. Two groups reported seeing WH in swampy areas and said that WH had a strong preference for water.

Farmers reported constant crop damage during the year to cassava or peanut plantations related to WH (with a decrease during the months from March to May), and crop damage related to BP was reported to increase during the wet season (when most of the crops are ripe).

### Association between WP–DP Interactions and ASF Occurrence

The proportion of farmers in the individual interviews reported suffering at least one ASF outbreak during the last year was 31.8% (*n* = 74), whereas 55.8% (*n* = 130) reported not being affected, and 10.7% (*n* = 25) did not know. During each ASF outbreak, farmers perceived that on average 65.7% of the animals died, 28.2% remained healthy throughout the outbreak, and 2.9% recovered after having shown clinical signs of the disease. The remaining 1.8% of the pigs strayed away or were sold. ASF outbreaks were reported significantly more often during the dry season than during the wet season (*T*-test, *p* = 0.017). Many of the respondents (*n* = 41, 17.6%) ate the carcasses of the DP that presumably died from ASF, whereas others left them in the field (*n* = 28, 12%) or sold them (*n* = 18, 7.7%). Spearman rank correlation coefficients (Rho) of housing system and WP sightings with ASF outbreaks were Rho_(ASF-free range)_ = 0.75 (*p* = 0.005); Rho_(ASF-confined pigs)_ = −0.73 (*p* = 0.006); Rho_(ASF-tethered pigs)_ = −0.75 (*p* = 0.005); Rho_(ASF-BP)_ = 0.84 (*p* < 0.001); and Rho_(ASF-WH)_ = 0.73 (*p* = 0.007).

**Figure 5:**
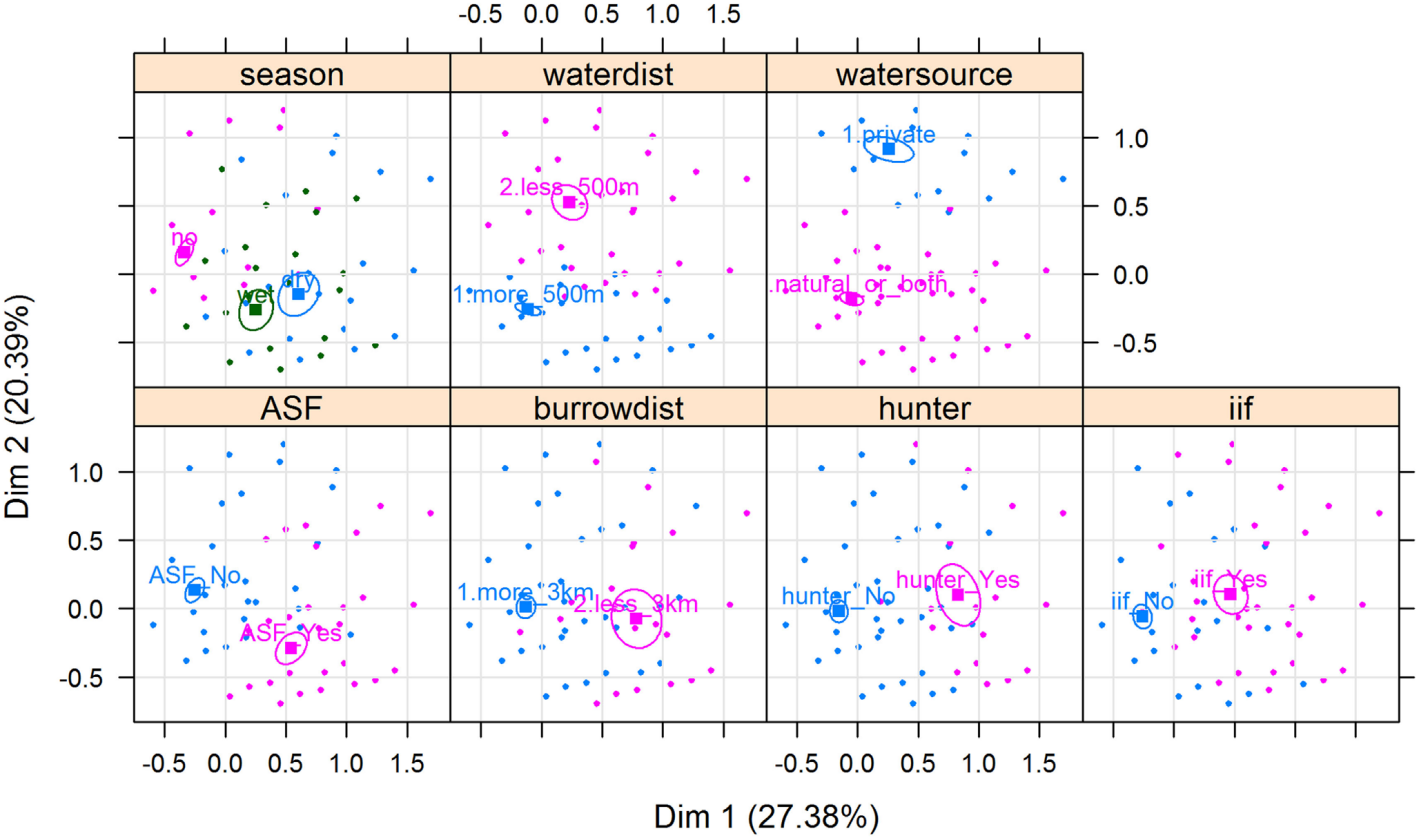
**Biplot of individuals and variable categories with confidence ellipses**. Labels for variable categories are displayed: watersource (private/natural_or_both); iif = indirect interaction (iif_Yes/iif_No); season = season of the ASF outbreak (dry/wet/no; “no” refers to no ASF outbreaks reported by the respondent); waterdist = HH distance from the pig’s water source (more_500 m/less_500 m); ASF = ASF outbreak (ASF_Yes/ASF_No); burrowdist = HH distance from a WH burrow (less than 3 km/more than 3 km); and hunter = respondent being a hunter (hunter_Yes/hunter_No).

**Figure 6:**
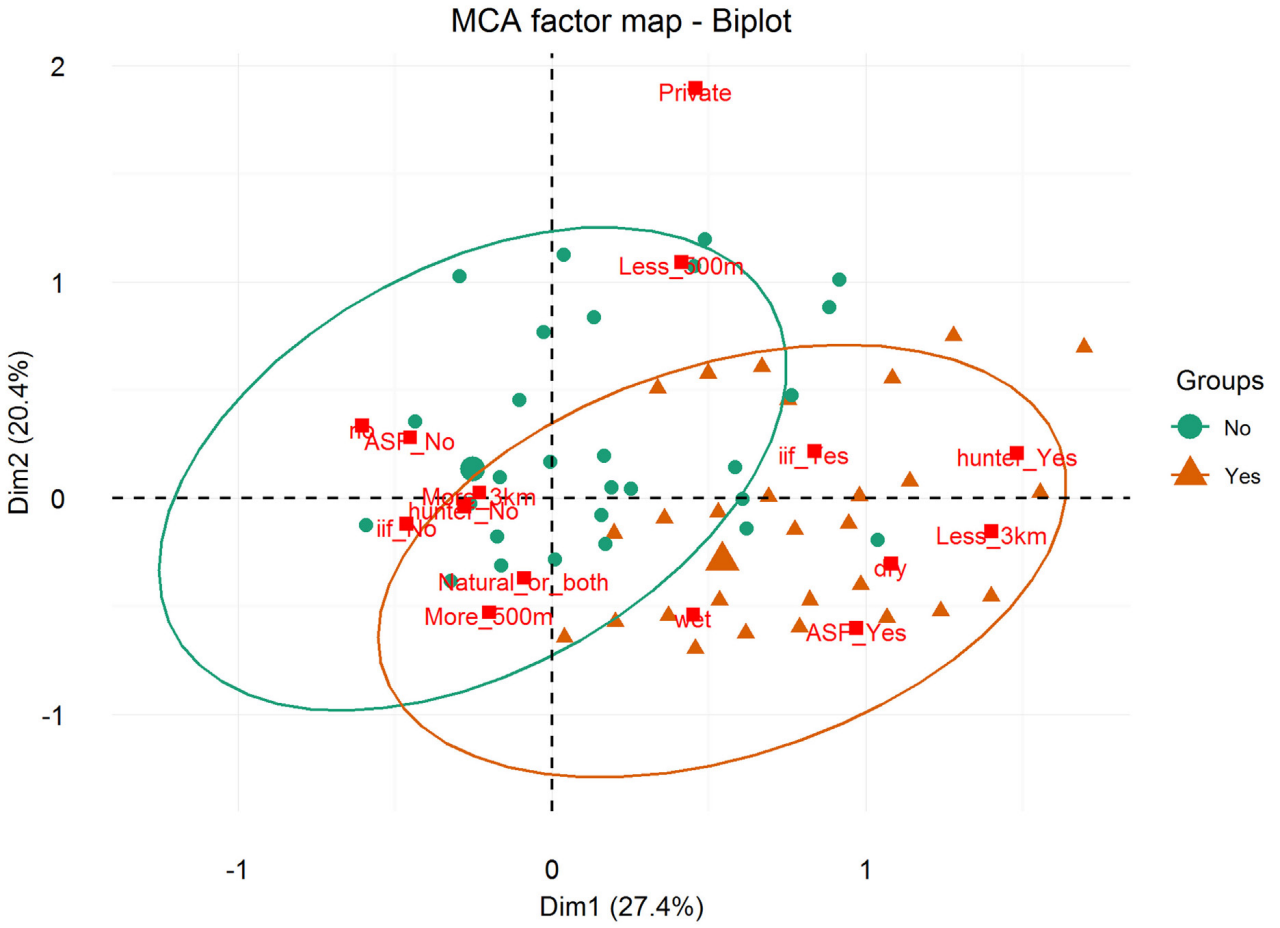
**Associations between ASF occurrence and several categorical variables depicted by a multiple correspondence analysis, from data collected during individual interviews carried out in pig owning household in Nwoya District, Uganda**. Coordinates of the categorical variables [namely, being a hunter, having indirect interactions with either warthog (WH) or bushpig, having a WH burrow less than 3 km from the HH, and season of ASF occurrence] are indicated by squares. Individuals with ASF occurrence is indicated by orange triangles, whereas those with ASF absence are indicated by green dots. Ellipses represent point concentrations. ASF.outbreak (ASF_Yes/ASF_No); Distance.to.Burrow = HH distance from a WH burrow (less than 3 km/more than 3 km); Being.Hunter = respondent being a hunter (hunter_Yes/hunter_No); Indirect.interaction (ii_Yes/ii_No); Season = season of the ASF outbreak (dry/wet/no; “no” refers to no ASF outbreaks reported by the respondent); Water.Distance = HH distance from the pig’s water source (more_500 m/less_500 m); and Water.Source (private/natural_or_both).

Results of the MCA show that the variables that most contributed to the definition of dimension one were “being a hunter = Yes” (15.91%), “ASF occurrence = Yes” (13.64%), “having a WH burrow less than 3 km from the HH = Yes” (12.68%), “having indirect interactions with either WP or BP = Yes” (11.38%), and “having had an outbreak during the dry season = Yes” (11.66%) (Figure 5). The variables that most contributed to the definition of dimension two were “water source = private” (35.96%) and “HH distance from the pig’s water source = less than 500 m” (23.83%) (Figures 5 and 6).

**Figure 7:**
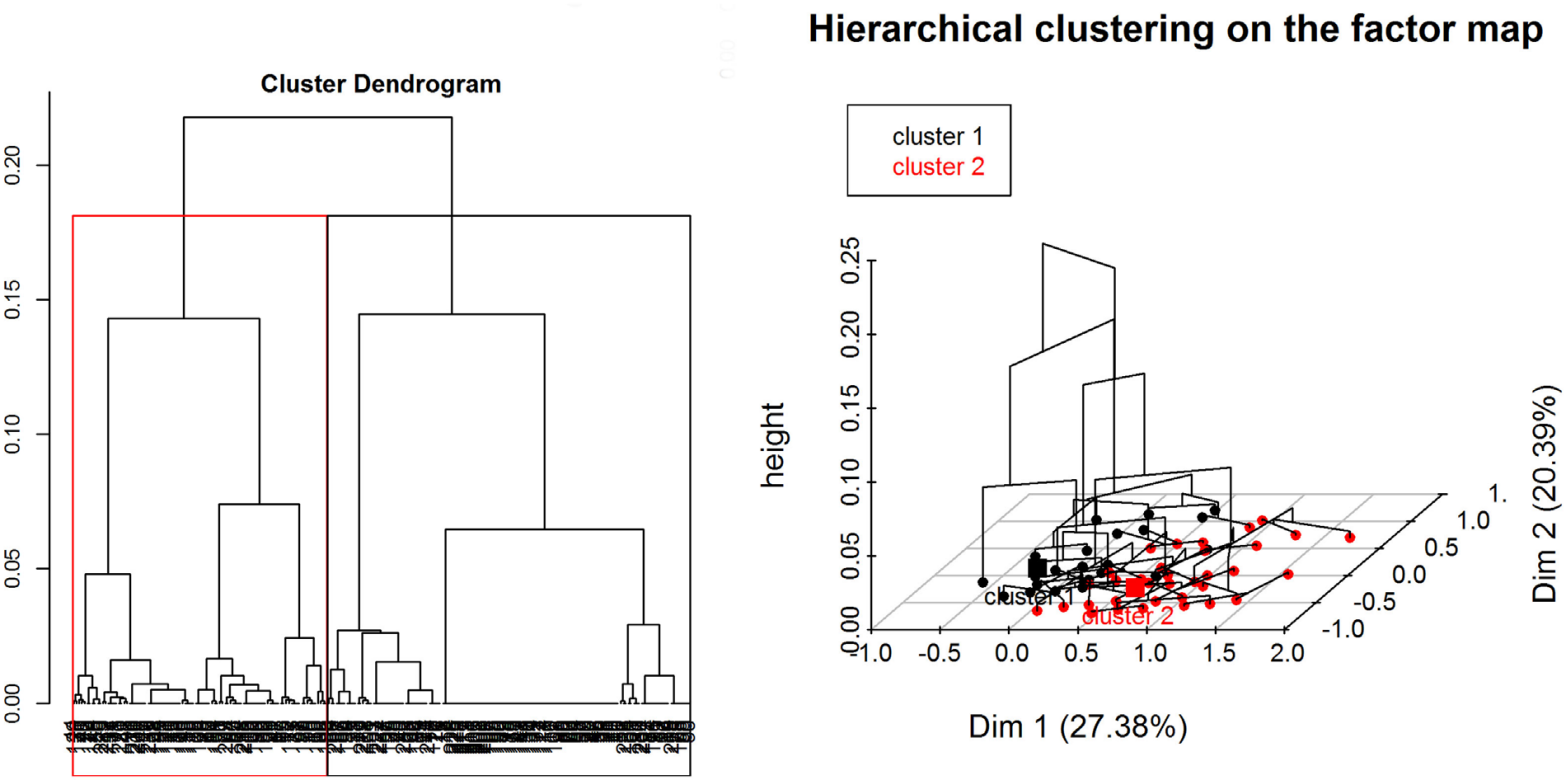
**Divisive hierarchical clustering analysis of categorical variables from data collected during individual interviews carried out in pig owning household in Nwoya District, Uganda, 2015**. Cluster one (black) and cluster two (red) (please refer to the online colored version) divide the data in HH without and with ASF occurrence, respectively.

Regarding the HCA, two main clusters were selected based on visual inspection of the overall appearance of the hierarchical tree and the visual representation of the individuals in the first two dimensions (Figure 7). A description of the HCA clusters based on the studied variables shows that the variables that influenced the most in characterizing the partition of the clusters (46) were “ASF occurrence” (yes/no), *p*-value <0.001; “season when the ASF outbreaks happened” (dry/wet/no – “no” refers to no ASF outbreak was reported by the respondent), *p*-value < 0.001; “HH distance from a WH burrow” (less than 3 km/more than 3 km), *p*-value <0.001; “respondent being a hunter” (yes/no), *p*-value <0.001; and “having indirect interactions with either WP or BP” (yes/no), *p*-value = 0.01. The most representative variables in each cluster are depicted in Table 3. As an example, we can see that 100% of the participants who suffered ASF outbreaks were in cluster two, and 90.24% of the participants who were in this cluster had suffered an ASF outbreak.

**Table 3:** **Description of the most representative variables of each cluster of a hierarchical cluster analysis of data collected regarding pig management during individual interviews implemented in household-owning pigs at Nwoya district, Uganda, in 2015**.

Variable	Variable outcome	% of the sample in the cluster?^a^	Percentage of category in cluster?^b^	Percent of category in sample^c^
**Cluster 1**				
ASF outbreak	No	94.97	100	68.24
Season when the ASF outbreaks happened	NA^d^	100	86	55.8
HH distance from a WH burrow	More than 3 km	70.5	93.38	85.84
Respondent being a hunter	No	69.39	90	84.12
**Cluster 2**				
ASF outbreak	Yes	100	90.24	31.76
Season when the ASF outbreaks happened	Dry season	84.3	52.44	21.89
	Wet season	75	47.56	22.32
HH distance from a WH burrow	Less than 3 km	70	28	14.16
Respondent being a hunter	Yes	59.46	26.83	15.88

*^a^Percentage of the sample in the cluster = % of individuals with the variable outcome in the study population who are in the cluster*.

*^b^Percentage of category in cluster = % of individuals in the cluster with the variable outcome*.

*^c^Percentage of category in sample = % of the variable in the study population*.

*^d^NA, not applicable, no ASF outbreak was reported by the respondent*.

## Discussion

This study provides unique, valuable information on the interactions between African WP (both WH and BP) and DP at the interface of an unfenced protected area in northern Uganda. To date, information about potential diseases other than ASF carried by wild African pigs is very scarce (8, 9) as these species have seldom been investigated from a veterinary perspective. Both WP species have been reported to become occasionally infected by bovine tuberculosis, trichinellosis, or foot-and-mouth disease (8, 9); moreover, BPs have also been found to become carriers of porcine parvovirus (48). Therefore, there are potential risks for transmission of these and other infectious pathogens from and to DPs through direct or indirect interactions. Thus, the collection of WP–DP contact information from local farmers is the first logical step to evaluate the potential risk of disease transmission in our study area.

Interestingly, DIs were not reported either in the individual interviews or the PRAs, suggesting that they are likely to be scarce or absent. Therefore, interspecies (DP–BP) mating and hybridization as anecdotally reported elsewhere in Africa (17) does not seem to occur in our study area. This lack of direct contact among BP and DP could be explained, at least in part, by the nocturnal and reserved behavior of BP, particularly during dry season, when the scarcity of ripped crops and of high grass/hiding spots may limit the incursions of BP into populated areas.

Contrary to our findings regarding the lack of DIs, indirect interactions were reported to be frequent (35.6% of respondents), particularly at water sources (i.e., swamps) and during the dry season (Figure 2). This period coincides with the time when most DP are on free range, when the WP hunting bag is larger, and when ASF outbreaks seem to be most prevalent (Figure 2). This suggests that water sources could be hotspots for both WP–DP and DP–DP direct or indirect contact (49), and thus for potential transmission of diseases. In this context, it is also worthwhile to note the positive correlation found between ASF occurrence and free-range housing system, WP sightings (Figure 2), and the dry season, although no further conclusions can be drawn at this stage.

Warthogs were reported to be easier to be seen and hunted during the dry season due to the shorter length of the grass. Nonetheless, crop damage related to WH was reported to remain constant during the year and crop damage related to BP was reported to increase during the wet season. Therefore, WP may be attracted to populated areas and consequently offer opportunities for interactions with DP to occur throughout the year. Studies assessing the potential interaction between WP and DP by telemetry are currently being conducted in the study area, and they will provide further insights into dynamics of WP–DP interactions in this area.

In this study, we used ASF as an example of transmissible diseases between WP and DP. Nevertheless, we must bear in mind that the epidemiology of ASF is complex and that DP can become infected through many different pathways, in addition to potential interactions with WP.

For example, the majority of ASFV outbreaks and spread within the DP populations in endemic settings, such as Northern Uganda, are most likely related to factors associated with human management practices and behavior in the DP value chain – and not to the presence of WP (16, 32). However, in the edge of protected areas, WP may play an important role for the persistence of ASFV and other diseases in the environment or for their introduction into naive populations of DPs.

The MCA supported the results of the descriptive analyses and highlighted the potential role of hunters as a link between WP and DP, facilitating spread of disease at the wildlife–livestock interface. Concurrently, the HCA indicated that individuals that suffered ASF can be clustered with those having a WH burrow less than 3 km from the HH and those being hunters. The majority of the self-declared hunters slaughtered WP in the field and left the offal in the hunting area. Although not a common practice, eight respondents (3.4% of the 233 respondents) reported feeding their DP with raw WP offal. These practices potentially entail a risk of transmission of ASFV and other diseases if the WP is infected (17). The temporal association between ASF outbreaks and a higher hunting activity during the dry season suggests that inappropriate management of WP offal could play a role in the transmission of ASF to DP. Further experimental studies are needed to appropriately investigate the association of ingestion of WP infected meat and ASF transmission in DP. Parker et al. (50) suggested that it is unlikely that DP could become infected with the ingestion of infected WH carcass offal. However, the use of different ASFV strains, WH age, and infective doses could play a role on ASF transmission and should therefore be studied.

In conclusion, preliminary information gathered through questionnaires suggests that DIs between WP and DP do not seem to occur at the northern interface of Murchison Falls National Park. However, indirect interactions between DP and WP are frequent and may pose an opportunity for disease transmission, particularly during the dry season, at water sources or through management of hunting carcasses. Moreover, crops, such as cassava or corn, may also be acting as points of attraction for WP, luring them closer to human settlements and thus, increasing the likelihood of WP–DP interactions. Further ecological studies with more sophisticated methods (telemetry and camera tarps) are currently being implemented to confirm the validity of questionnaires in gathering valuable information about those interactions in this area. In the meantime, outreach and education activities to minimize DP–WP indirect contacts (e.g., discourage of free-ranging systems for DP or limit the access to disposed WP carcasses) will likely help to reduce the risk of the potential dissemination of pathogens between wild and DP species.

## Author Contributions

All authors participated in the design of the study. DC coded the questionnaire into KoBo toolbox online platform. EK and EC collected the data during fieldwork. BM-L and EK performed the analysis. EK wrote the manuscript. EK, FJ, BM-L, EC, CM, and KS participated in drafting the manuscript or revising it critically for important intellectual content.

## Conflict of Interest Statement

The authors declare that the research was conducted in the absence of any commercial or financial relationships that could be construed as a potential conflict of interest.

## Footnotes

^1^http://epitools.ausvet.com.au/

^2^http://www.kobotoolbox.org/
