# Supplementary material for: Wild and Domestic Pig Interactions at the Wildlife–Livestock Interface of Murchison Falls National Park, Uganda, and the Potential Association with African Swine Fever Outbreaks
Source: Front Vet Sci. 2016 Apr 14;3:31. doi: 10.3389/fvets.2016.00031 (PMC4831202; doi:10.3389/fvets.2016.00031)
Supplement: Supplementary file 2 [file datasheet_2.pdf]

# Survey Uganda

**I confirm that: (i) I have understood the explanation of the research as set out in the project information sheet. (ii) I have had the opportunity to ask questions about the research and I am happy that my questions have been answered. (iii) I am happy to answer the questions that the research team asks me. I know that if I do not want to answer any question I can say "I do not want to answer that". (iv) I know that my answers to questions will be stored and used in ways that keep my name and the exact location of my farm private and confidential. (v) I understand that the research team will tell veterinary authorities about what the whole research project finds out, but my personal data and answers will remain confidential. (vi) The researchers can use photographs of my farm or family in talks or articles about the research.**

*

○ OK

**Introduction**

**Gender**

○ M

○ F

**Location**

GPS coordinates can only be collected when outside.

latitude (x.y °) longitude (x.y °) altitude (m) accuracy (m)

**Subcountry**

**Perish**

**Village**

**Household demographics and characteristics**

**» 1.How many of the following do you have in your homestead at the moment?**

**Type of pig**

- ◯ Breeding saw
- ◯ Breeding boars
- ◯ Growers
- ◯ Piglets
- ◯ None

**Number of pigs**

**» 2.Which housing system(s) do you use throughout the year?**

**Housing system**

- ◯ Free range
- ◯ Tethering
- ◯ House

**Month**

- ☐ January
- ☐ February
- ☐ March
- ☐ April
- ☐ May
- ☐ June
- ☐ July
- ☐ August
- ☐ September
- ☐ October
- ☐ November
- ☐ December
- ☐ Equally throughout the year

**3. Last year, did you ever notice the presence of soft ticks on your farm or surroundings?**

- ◯ Yes
- ◯ No

**3.1. Where did you see them?**

- ◯ In holes or crevices in your home
- ◯ In holes or crevices in your pig sty
- ◯ On wild pig’s burrows
- ◯ Other

**Please specify**

---

**4. How do your pigs access water?**

- ◯ Private water trough - only for your pigs
- ◯ Natural water trough (river, lakes, ponds)- shared with other pigs or wildlife
- ◯ Both natural and private
- ◯ Other

**Please specify**

---

**5. How far away from your household is that water source?**

- ◯ <500 m
- ◯ 500 m - 2km
- ◯ >2 km

**6. Are you also a wild pig hunter?**

- ◯ Yes
- ◯ No

**» 6.1. Specify the average number of hunted wildpigs during last year according to the period of the year when you hunted them.**

**Type of pig**

- ◯ Bushpig
- ◯ Warthog

**Month**

- ◯ January
- ◯ February
- ◯ March
- ◯ April
- ◯ May
- ◯ June
- ◯ July
- ◯ August
- ◯ September
- ◯ October
- ◯ November
- ◯ December

**Number**

**6.2. Where do you slaughter the wild pig carcasses you hunt?**

- ☐ At the hunting area
- ☐ At home
- ☐ At the market
- ☐ Other

**Please specify**

______________________________

**6.3. After slaughtering the wild pigs, what do you do with the left-overs?**

- ☐ Burn them
- ☐ Bury them
- ☐ Leave them on the field
- ☐ Feed them to the dogs
- ☐ Other

**Please specify**

______________________________

**6.4. Do your pigs eat the raw left overs of your hunted wild pigs?**

- ◯ Yes
- ◯ No

**Wild pig’s indirect interactions**

**7. In the last 12 months, have you seen any wild pigs in your village area?**

- ◯ Yes
- ◯ No
- ◯ I've only heard them or seen a trail

**» 7.1 On average, how close to your household do they come?**

**Type of pig**

◯ Bushpig ◯ Warthog

**Distance**

- ◯ Less than 10 m
- ◯ 10-50 m
- ◯ 50-500 m
- ◯ > 500 m

**» 7.2. On average, how many wild pigs did you see in your village last year?**

**Type of pig**

◯ Bushpig ◯ Warthog

**Number**

**» 7.3. When do you usually see them?**

**Type of pig**

- ◯ Bushpig
- ◯ Warthog

**Month**

- ☐ January
- ☐ February
- ☐ March
- ☐ April
- ☐ May
- ☐ June
- ☐ July
- ☐ August
- ☐ September
- ☐ October
- ☐ November
- ☐ December
- ☐ Equally throughout the year

**» 7.4. Where have you seen them more often?**

**Type of pig**

- ◯ Bushpig
- ◯ Warthog

**Location**

- ☐ Cassava fields
- ☐ Onion fiels
- ☐ Mangoes areas
- ☐ Maize fields
- ☐ Ground nut fields
- ☐ Bush
- ☐ Puddle
- ☐ Lake
- ☐ River
- ☐ Other

**Please specify**

______________________________

**Wild pig's direct interactions**

**8. In the last 12 months, have you seen wild pigs being together (same location within 100 m at the same time) with domestic pigs in your village?**

- ○ Yes
- ○ No

**» 8.1. Where did those "contacts" happened more often?**

**Type of pig**

○ Bushpig ○ Warthog

**Location**

- ☐ Cassava fields
- ☐ Onion fiels
- ☐ Mangoes areas
- ☐ Maize fields
- ☐ Ground nut fields
- ☐ Bush
- ☐ Puddle
- ☐ Lake
- ☐ River
- ☐ Other

**» 8.2. For "Bushpig", please specify which type of "contact" and how much time the animals spent together.**

**Type of interaction**

- ◯ Mating - courtship
- ◯ Fighting
- ◯ Eating together
- ◯ Drinking together

**Number of times per year**

- ◯ 1 - 5
- ◯ 6 - 10
- ◯ > 10

**Duration**

- ◯ < 10 min
- ◯ < 1 h
- ◯ 1 - 3 h

**» 8.2. For “Warthog”, please specify which type of “contact” and how much time the animals spent together.**

**Type of interaction**

- ◯ Mating - courtship
- ◯ Fighting
- ◯ Eating together
- ◯ Drinking together

**Number of times per year**

- ◯ 1 - 5
- ◯ 6 - 10
- ◯ > 10

**Duration**

- ◯ < 10 min
- ◯ < 1 h
- ◯ 1 - 3 h

**» 8.3. For “Bushpig”, during last year, when did those “contacts” happen more often?**

**Type of interaction**

- ◯ Mating - courtship
- ◯ Fighting
- ◯ Eating together
- ◯ Drinking together

**Month**

- ☐ January
- ☐ February
- ☐ March
- ☐ April
- ☐ May
- ☐ June
- ☐ July
- ☐ August
- ☐ September
- ☐ October
- ☐ November
- ☐ December
- ☐ Equally throughout the year

**» 8.3. For “Warthog”, during last year, when did those “contacts” happen more often?**

**Type of interaction**

- ○ Mating - courtship
- ○ Fighting
- ○ Eating together
- ○ Drinking together

**Month**

- ☐ January
- ☐ February
- ☐ March
- ☐ April
- ☐ May
- ☐ June
- ☐ July
- ☐ August
- ☐ September
- ☐ October
- ☐ November
- ☐ December

**» 9. On average, do you remember the distance between your pigs and wild pigs when you saw the “contacts”?**

**Type of pig**

- ○ Bushpig
- ○ Warthog

**Distance**

- ○ < 1 m
- ○ 1 - 10 m
- ○ 11 - 50 m
- ○ 51 - 100 m
- ○ > 100 m

**10. If mating “contacts” occurred with bushpigs, in which way did it happen?**

- ☐ Domestic female mating with male bushpigs
- ☐ Domestic male mating with female bushpigs

**10.1. If they gave birth (after mating with bushpigs), how many piglets did you get last year?**

**10.2. What did you do with this offspring?**

- ☐ I used them for own consumption
- ☐ I sold them as piglets
- ☐ I sold them as growers
- ☐ I kept them as breeders
- ☐ Other

**Please specify**

______________________________

**11. If fighting “contacts” occurred, were any pigs wounded?**

- ○ Yes
- ○ No

**11.1. Why did they fight for?**

- ☐ Mating
- ☐ Food
- ☐ Water
- ☐ I don't know

**12. If drinking “contacts” occurred, how far away from your household is that water source?**

- ○ < 500 m
- ○ 500 m - 2 km
- ○ > 2 km

**Perception and management of direct interactions**

**13. Are “contacts” with wild pigs a nuisance to you?**

- ○ Yes
- ○ No

**13.1. Please specify why**

- ☐ Fear of transmission of disease to my pigs
- ☐ Crop damage
- ☐ Other

**Please specify**

---

**14. Have you taken any measure to specifically avoid “contacts” between your pigs and wild pigs?**

- ◯ Yes
- ◯ No

**14.1. Specify please**

- ☐ Wire fence
- ☐ Banana leaves fence
- ☐ Dogs
- ☐ Keep the pigs housed
- ☐ Keep the pig tethered
- ☐ Other

**Please specify**

---

**15. Have you ever seen any warthog’s burrow close to your village?**

- ◯ Yes
- ◯ No

**15.1. Please, specify where**

- ◯ < 1 km away
- ◯ 1 - 3 km away
- ◯ > 3 km

**Disease information**

**16. During the last 12 months, have you seen a lot of your pigs dying at once?**

- ◯ Yes
- ◯ No

**16.1. If yes, do you suspect it was caused by African swine fever?**

- ◯ Yes
- ◯ No
- ◯ I don't know

**16.2. When did the deaths happen?**

- ☐ January
- ☐ February
- ☐ March
- ☐ April
- ☐ May
- ☐ June
- ☐ July
- ☐ August
- ☐ September
- ☐ October
- ☐ November
- ☐ December
- ☐ Equally throughout the year

**» 16.3. How many pigs...**

**How many pigs...**

- ◯ ...did you have before the outbreak?
- ◯ ...died during the outbreak?
- ◯ ...recovered after showing signs of disease?
- ◯ ...remained healthy throughout the whole outbreak?

**Number of animal**

**» 16.4. During the outbreak, if you had offspring from bushpig mating,...**

**How many of them...**

- ◯ ...died?
- ◯ ...recoverd after showing signs of disease?
- ◯ ...remained healthy throughout the whole outbreak?

**Number of animal**

**16.5. What did you do with the carcass of your dead pigs?**

- ☐ Burn it
- ☐ Buried it
- ☐ Left it on the field
- ☐ Fed it to the dogs
- ☐ Eat it
- ☐ Sell it
- ☐ Other

**Please specify**

______________________________

**16.6. Have you ever seen wild pigs around these carcasses of your domestic pigs?**

- ◯ Yes
- ◯ No
