# Supplementary material for: Wild and Domestic Pig Interactions at the Wildlife–Livestock Interface of Murchison Falls National Park, Uganda, and the Potential Association with African Swine Fever Outbreaks
Source: Front Vet Sci. 2016 Apr 14;3:31. doi: 10.3389/fvets.2016.00031 (PMC4831202; doi:10.3389/fvets.2016.00031)
Supplement: Supplementary file 3 [file datasheet_3.docx]

**Understanding the dynamics and spread of African swine fever virus at the wildlife-livestock interface: A participatory rural appraisal following a closed questionnaire on wildlife-livestock interactions**

## Invitation to participate

We are conducting a research project where we investigate the interactions between your domestic pigs and wild pigs in order to understand the relationship between African swine fever virus, domestic and wild pig. Our final aim is to prevent pig diseases in your community and, in this way, to keep your pigs healthier. You were randomly selected to form part on the first part of our study – the questionnaire about wild pigs’ interactions with your pigs. This time we are inviting you to participate in the second part of our research. In this section of our research we want to ask you questions about the seasonality of different events and your perception of certain factors related with wild pigs and African swine fever in your community. The questions we will ask you are to be replied as a group. As with the questionnaires, if there is a question that you do not want to answer, you can say “I do not want to answer that question”. This exercise will take about 2 hours and 30 minutes. Once we finish with the group questions, we will present and discuss the signs your pigs may show when infected with African swine fever virus, the way it can be transmitted and will recommend ways to keep your pigs safe from getting the disease.

We may also want to make some photographs of your farm and family. We might want to use some of these photographs in talks or articles about the research, but without disclosing any identities.

## Your consent to participate

You do not have to agree to participate in this research. You can say ‘No’ now, or you can say ‘No’ after you understand more about the research. If you agree to participate we will keep your information private, but also ask you to sign a consent form.

## Organizations involved in this research

The project is being undertaken by researchers from Uganda (Makerere University) Sweden (SLU), France (CIRAD), and USA (University of California).

## We will keep your information private

The information you provide to us will be kept private and confidential. Only the fieldwork leader Esther Kukielka and senior members of the research team authorized by Esther will be able to look up your name. When we use the information about you in our research reports, we will not use your name or the exact location of your farm.

## Benefits and risks to participants

We are doing this research to help the understanding of the interactions that may exist between your domestic pigs and wild pig and the consequences of this with regards to African swine fever transmission. These are big and complicated problems. This research will not provide all the answers that are needed and will not stop the risk of your pigs getting sick. But we will give you advice about the best, simple ways that we know of, to keep your pigs healthy by sharing our results with the veterinary authorities of Nwoya and local leaders. We will avoid coming in contact with the pigs so that we don’t bring diseases from other places to your farm.

## Contacts for questions or problems about the research

If you have questions about the research after today, you should contact one of these people on the research team:

Fieldwork leader: Esther Kukielka: XXXXXXXXXX

Farmer survey leader: Tony Aliro: XXXXXXXXXX
