# Supplementary material for: Wild and Domestic Pig Interactions at the Wildlife–Livestock Interface of Murchison Falls National Park, Uganda, and the Potential Association with African Swine Fever Outbreaks
Source: Front Vet Sci. 2016 Apr 14;3:31. doi: 10.3389/fvets.2016.00031 (PMC4831202; doi:10.3389/fvets.2016.00031)
Supplement: Supplementary file 4 [file datasheet_4.docx]

Date____________________________

Starting time_____________________ Finish time___________________________________

Name of Perish___________________________ Subcounty_______________________

Total number of participants in the group:___________________________________________________

Facilitator_____________________________________________________________________________

Note-taker____________________________________________________________________________

Objective: The objective is to gather information from pig farmers regarding the interaction between their pigs and wild pigs and the farmer’s perception of the impact and disease related risks posed by the latest.

## Introduction by the facilitator

1. Welcome the participants. Have one of them open with a word of prayer.
2. Introduce yourself and the team.
3. Have them introduce themselves.
4. Setting the scene: Introduce the project, the consent form, highlighting the objectives and the important role of the participants in meeting the objectives*.*
5. Ask for consent to use cameras.
6. **Seasonality of several factors**

**Purpose:** This activity aims at understanding the seasonality of several factors of interest. Likewise, it will help to understand the significance of the “dry” and “wet” season concepts amongst farmers (*during the questionnaire, several respondents described the year on such terms instead of discrete months’ names*). Further, it serves as a warm up exercise to start the journey.

**Tool:** Seasonal calendar.

**Facilitator**: The seasonality of rainfall and several activities or occurrences is depicted by indicating the timing of occurrence or scoring occurrence in relation to the months of the year, using 100 counters for each factor.

|  | Months | | | | | | | | | | | |
| --- | --- | --- | --- | --- | --- | --- | --- | --- | --- | --- | --- | --- |
| Event | J | F | M | A | M | J | J | A | S | O | N | D |
| Local name of season |  |  |  |  |  |  |  |  |  |  |  |  |
| Rainfall |  |  |  |  |  |  |  |  |  |  |  |  |
| Bushpig’s hunting season* |  |  |  |  |  |  |  |  |  |  |  |  |
| Warthog’s hunting season* |  |  |  |  |  |  |  |  |  |  |  |  |
| Presence of bushpig in the community ^Δ^ |  |  |  |  |  |  |  |  |  |  |  |  |
| Presence of warthog in the community ^Δ^ |  |  |  |  |  |  |  |  |  |  |  |  |
| Crop damage caused by wildlife |  |  |  |  |  |  |  |  |  |  |  |  |

* Do they *attempt* to hunt throughout the year? Or do they focus more during a specific time of the year? Notice the word “attempt”. We’re not talking only about “when” they manage to hunt them (that we already know from the questionnaires).

^Δ^ As a method of triangulation used with the questionnaires

1. **Seasonality of crop damaged**

**Purpose:** This activity aims at understanding the seasonality of crop damage caused by bushpig and warthog.

**C. Tool:** Seasonal calendar

**Facilitator**: The seasonality of crop damage is depicted by indicating the timing of occurrence in relation to the months of the year, using 100 counters for each factor.

| Animal | Months | | | | | | | | | | | |
| --- | --- | --- | --- | --- | --- | --- | --- | --- | --- | --- | --- | --- |
|  | J | F | M | A | M | J | J | A | S | O | N | D |
| Bushpig |  |  |  |  |  |  |  |  |  |  |  |  |
| Warthog |  |  |  |  |  |  |  |  |  |  |  |  |

1. **Direct interactions**

**Purpose:** This activity aims at inquiring about the possible sighting of direct interactions (same location within 100 m at the same time) between wild pigs and domestic pigs *(no limit of time, the direct interaction could have been seen ten years ago).*

**Tool:** Hand count and listing

**Facilitator**: The number of farmers having seen direct interactions will be counted as per lifted hands (one point per farmer maximum). If direct interactions have been seen, the facilitator will probe for answers to the following questions:

1. If they have not seen a direct interaction, have they ever heard of it from someone else?

2. How many people have seen direct interactions happening?

3. Which species (bushpig, warthog, or both)?

4. How many times have you seen it happening?

5. What time of the day did it happened (sunrise, day, sunset, night?

6. In which month did the direct interaction happen?

7. Where did the direct interaction happen? *For example, in the swamp, in the cassava fields or in the bush.*

8. Which type of contact happened? *For example, they were mating together, or eating together, or drinking together, or fighting*.

9. How long did the animals remain together?

10. How close were they? How many meters were there between the animals?

11. Have they ever seen domestic sows mating with male bushpigs?

12. Have they ever heard from someone else about domestic sows mating with male bushpigs? *(In this case, they have not seen it themselves, but they have heard of that happening)*

13. Have they even seen or heard of a domestic sow getting crossbred pigs as result of mating with bushpigs?
